# Impact of St. John’s wort extract Ze 117 on stress induced changes in the lipidome of PBMC

**DOI:** 10.1186/s10020-023-00644-3

**Published:** 2023-04-07

**Authors:** Hendrik Bussmann, Swen Bremer, Hanns Häberlein, Georg Boonen, Jürgen Drewe, Veronika Butterweck, Sebastian Franken

**Affiliations:** 1 Max Zeller Söhne AG, Seeblickstrasse 4, 8590 Romanshorn, Switzerland; 2 Institute of Biochemistry and Molecular Biology, Medical Faculty, University of Bonn, Nussallee 11, 53115 Bonn, Germany

**Keywords:** Depression, *Hypericum perforatum*, Cortisol, Stress, Lipidomics, Membrane fluidity

## Abstract

**Background:**

Membrane lipids have an important function in the brain as they not only provide a physical barrier segregating the inner and outer cellular environments, but are also involved in cell signaling. It has been shown that the lipid composition effects membrane fluidity which affects lateral mobility and activity of membrane-bound receptors.

**Methods:**

Since changes in cellular membrane properties are considered to play an important role in the development of depression, the effect of St. John’s wort extract Ze 117 on plasma membrane fluidity in peripheral blood mononuclear cells (PBMC) was investigated using fluorescence anisotropy measurements. Changes in fatty acid residues in phospholipids after treatment of cortisol-stressed [1 μM] PBMCs with Ze 117 [10–50 µg/ml] were analyzed by mass spectrometry.

**Results:**

Cortisol increased membrane fluidity significantly by 3%, co-treatment with Ze 117 [50 µg/ml] counteracted this by 4.6%. The increased membrane rigidity by Ze 117 in cortisol-stressed [1 μM] PBMC can be explained by a reduced average number of double bonds and shortened chain length of fatty acid residues in phospholipids, as shown by lipidomics experiments.

**Conclusion:**

The increase in membrane rigidity after Ze 117 treatment and therefore the ability to normalize membrane structure points to a new mechanism of antidepressant action of the extract.

**Supplementary Information:**

The online version contains supplementary material available at 10.1186/s10020-023-00644-3.

## Background

Depression is characterized by various symptoms such as depressed mood, fatigue, loss of appetite and self-esteem (2013), lack of concentration and suicidal thoughts. According to the World Health Organization, the lifetime prevalence of depression is estimated to be over 20%, and it is expected to be the leading form of disability by the year 2030 (Vos et al. 2015). Due to the heterogenous symptoms of depression, diagnosing and treating the illness is a major challenge. It is further complicated by the fact that the exact pathophysiology of depression still remains unclear. Biochemical biomarkers which would allow an objective diagnosis of the disease are still not available (Wager and Woo 2017). Several hypotheses exist explaining the pathogenesis of depression, the most prevalent one is the monoamine hypothesis, indicating that depression is caused by an imbalance of serotonin, norepinephrine and dopamine in the brain (Schildkraut 1965). However, the limitation of this hypothesis is that it cannot explain why it takes several weeks of treatment before an antidepressant effect becomes evident, although neurotransmitter levels increase shortly after therapy was initiated (Berton and Nestler 2006). Since meanwhile there is sufficient evidence that the monoamine hypothesis of depression is inaccurate (Moncrieff et al. 2022), further hypotheses were formed. Another hypothesis implicates an involvement of the hypothalamic–pituitary–adrenal (HPA) axis in the pathophysiology of depression (van Bodegom et al. 2017). Hyperactivity of the HPA axis as indicated by increased levels of corticotropin-releasing factor (CRF) in the brain accompanied by elevated plasma cortisol levels is a key finding in approximately 40–60% of depressed patients (Barden 2004; Keller et al. 2017). During successful antidepressant treatment a normalization of the HPA system occurs (Barden 2004).

It has been shown that increased glucocorticoid levels may impact brain membrane lipid composition, e.g. by increasing ceramide levels and concomitantly decreasing sphingomyelin levels in prefrontal cortex and hippocampus of rats (Müller et al. 2015). Membrane lipids not only provide a barrier between intra- and extracellular compartments of a cell but also determine the localization and function of membrane proteins, thus, influencing vesicle synthesis, neurotransmitter release and cell signaling (Miranda and Oliveira 2015). Since lipids play an important role in brain function, their involvement in stress-related disorders such as depression is not unexpected and it is meanwhile recognized that altered membrane lipid compositions in patients correlate with the occurrence of depressive disorders (Müller et al. 2015). For example, it has been shown that due to increased oxidative stress lipid peroxidation is increased in depression resulting in an altered lipid profile and lipid metabolism in the brain (Stirton et al. 2021).

Extracts of St. John’s wort (SJW, *Hypericum perforatum* L., Hypericaceae) are successfully used to treat mild to moderate forms of depression (Linde et al. 2008). Although extensively researched in the last two decades (Schmidt and Butterweck 2015), there are still many open questions regarding the mechanism of action of SJW. Besides an interaction with various neurotransmitter receptors (Butterweck 2003), neuroendocrine studies showed an involvement of SJW in the regulation of genes that control HPA axis function after chronic stress (Butterweck et al. 2001a, b, 2004; Verjee et al. 2018).

Since membrane lipid alterations seem to play an important role in the development of depressive disorders, changes in cellular lipids might present the missing link not only to the various hypotheses of depression but might also explain the mechanism of action of SJW.

Thus, in the present work changes in membrane fluidity were assessed using peripheral blood mononuclear cells (PBMC) to get an insight if the SJW extract Ze 117 interferes with cellular lipid composition. To further investigate the mechanism of action of Ze 117, a comprehensive lipidomic analysis was performed. Co-incubation of cortisol with Ze 117 was done to investigate whether the extract counteracts a possible cortisol-stress-induced change in the lipid composition of PBMCs. Since the saturation and chain length of the fatty acid moieties have a strong impact on membrane fluidity, the average chain length, and the average number of double bonds of several phospholipid classes were assessed in addition. The study was conducted at PBMC to establish a feasible tool that could potentially contribute to the monitoring of the pathogensis and treatment of depression.

## Materials and methods

### Drugs and reagents

A quantified extract from *Hypericum perforatum* low in hyperforin (Ze 117; DER 4–7:1; extraction solvent ethanol 57.9% (V/V); Batch number V803900) with 0.26% hypericin and < 0.2% hyperforin, was manufactured according to Ph. Eur. EP 9.3/1874 and provided by Max Zeller and Söhne AG, Romanshorn, Switzerland. The spissum extract Ze 117 was dissolved in 50% ethanol obtaining a stock solution with a concentration of 50 mg/ml. Before initiating experiments, the extract was further diluted with PBS to the final testing concentrations. The ethanol concentration in the cell medium did not exceed 0.05%. A HPLC chromatogram is given in Additional file 2: Fig. S1.

DPH (1,6-diphenyl-1,3,5-hexatriene) and cortisol were purchased from Sigma (Taufkirchen, Germany). For preparing the DPH stock solution, DPH was dissolved in DMSO (10 mM). The DMSO concentration during fluorescence anisotropy experiments did not exceed 0.025%).

The cortisol stock solution had a concentration of 1 mM dissolved in methanol. The methanol concentration during the assay did not exceed 0.1%.

#### Cell culture

Frozen aliquots of 2 × 10^7^ peripheral blood mononuclear cells (PBMC, ATCC, Manassas, Virginia, USA) were thawed in a 37 °C water bath and rinsed with 1 ml ice cold HBSS supplemented with 20% fetal calf serum (FCS, Gibco by Fisher Scientific, Bremen, Germany). The suspension was transferred into a conical tube and was centrifuged at 4 °C at 300×*g* to a cell pellet. The pellet was washed with phosphate buffered saline (PBS, Gibco by Fisher Scientific, Bremen, Germany) and then resuspended in 10 ml RPMI medium (Gibco by Fisher Scientific, Bremen, Germany) supplemented with 20% FCS, 2 mM l-glutamine, 100 units/ml penicillin and 100 µg/ml streptomycin. PBMC were cultured in cell culture flasks with vented caps (Sarstedt, Nürnberg, Germany) for fluorescence anisotropy or for lipidomics in 75 cm^2^ culture flask (Corning, New York City, New York, USA) at 37 °C in a humid atmosphere containing 5% CO_2_. All steps of cell cultivation and processing were performed under sterile conditions using only prewarmed (37 °C) solutions.

For fluorescence anisotropy experiments incubation of PBMC was performed for at least 4 days using cell culture medium enriched with 25 µg/ml Ze 117 and 1 µM cortisol. To each series of experiments a corresponding untreated control was prepared. Solvents were individually added to each condition and to the untreated control to reach a uniform solvent concentration of max. 0.1% MeOH and 0.1% EtOH. After 4 days fluorescence anisotropy measurements were performed. Therefore, cell suspension was transferred into a conical tube and was centrifuged at room temperature at 300×*g* to pellet cells. The pellet was resuspended in HBSS containing 2.5 µM DPH. Afterwards, cells were incubated for another 20 min at 37 °C in a humid atmosphere containing 5% CO_2_.

For lipidomics experiments cells were also incubated for 4 days. On the first day cortisol or Ze 117 was added to the medium in the respective culture flask according to the following scheme, resulting in five different conditions, namely a control condition, 1 µM cortisol, 1 µM cortisol plus 10 µg/ml Ze 117, 1 µM cortisol plus 25 µg/ml Ze 117, and 1 µM cortisol plus 50 µg/ml Ze 117. Concentrations were chosen based on previous experiments (Keksel et al. 2019) Solvents were individually added to each condition and to the untreated control, to reach a uniform solvent concentration. The cells were each washed twice in PBS and centrifuged. Then, the cells were resuspended in PBS, resulting in a cell concentration of 3000 cells/μl. The cell suspensions were transferred into 1.5 ml Eppendorf vessels and shock frozen using liquid nitrogen. Until shipment the cells were stored at − 80 °C.

#### Fluorescence anisotropy measurements on cells in suspension

The fluorescence anisotropy measurements were performed with a PerkinElmer LS55 fluorescence spectrometer (L-configuration, PerkinElmer, Waltham, Massachusetts, USA). The fluorescence spectrometer was equipped with a circulation thermostat with stirring unit, which heated the cell suspension to a constant 37 °C during the measurements and ensured that the cells were evenly distributed. The absorption polarizer was fixed to vertical and the emission polarizer could be adjusted variably vertically or horizontally. Because of that the possible polarization filter configurations were vertical and vertical (vv) or vertical and horizontal (vh). For the measurement with DPH the excitation wavelength was set to ʎex = 360 nm. The emission wavelength was set to ʎem: 430 nm.

The measurements started 5 min after placing the cuvette in the cuvette holder of the spectrometer. This ensures that the temperature was equalized by the heating device. For each experiment 10 individual anisotropy measurements were performed, measuring flourescence intensities I vv and I vh.

Anisotropy r was calculated as follows:

1 $$Anisotropy\;r= \frac{I\;vv - I\;vh}{I\;vv + 2 \cdot I\;vh }$$

**Fig. 1:**
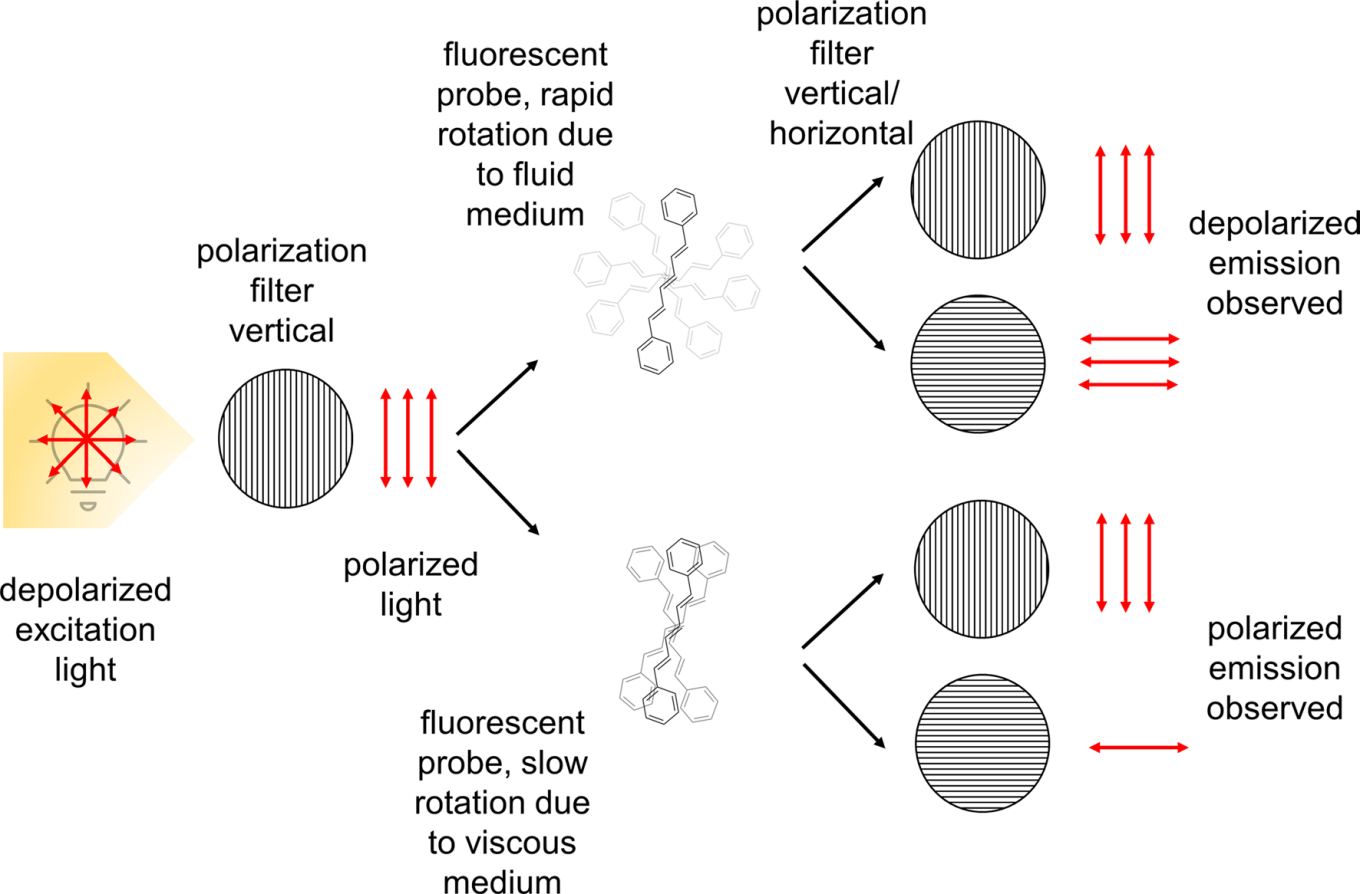
Principle of fluorescence anisotropy assay. The fluorescent probe DPH is excited by light which is polarized by a polarization filter in vertical position. When the probe is in a fluid surrounding, e.g. a membrane, it rotates and the emitted light is depolarized. When the membrane gets more viscous the rotation is attenuated and the emitted light is more polarized. The fluorescence intensity is measured by applying parallel and perpendicular polarization filters to the excitation light plane

The scheme of fluorescence anisotropy measurements is given in Fig. 1.

### Lipidomics

#### PBMC lipids analyzed by mass spectrometry

Mass spectrometry-based lipid analysis was performed by Lipotype GmbH (Dresden, Germany) as described by Sampaio et al. (2011). Lipids were extracted using a two-step chloroform/methanol procedure (Ejsing et al. 2009). Samples were spiked with internal lipid standard mixture containing: cardiolipin 14:0/14:0/14:0/14:0 (CL), ceramide 18:1; 2/17:0 (Cer), diacylglycerol 17:0/17:0 (DAG), hexosylceramide 18:1; 2/12:0 (HexCer), lyso-phosphatidate 17:0 (LPA), lyso-phosphatidylcholine 12:0 (LPC), lyso-phosphatidylethanolamine 17:1 (LPE), lyso-phosphatidylglycerol 17:1 (LPG), lyso-phosphatidylinositol 17:1 (LPI), lyso-phosphatidylserine 17:1 (LPS), phosphatidate 17:0/17:0 (PA), phosphatidylcholine 17:0/17:0 (PC), phosphatidylethanolamine 17:0/17:0 (PE), phosphatidylglycerol 17:0/17:0 (PG), phosphatidylinositol 16:0/16:0 (PI), phosphatidylserine 17:0/17:0 (PS), cholesterol ester 20:0 (CE), sphingomyelin 18:1;2/12:0;0 (SM), triacylglycerol 17:0/17:0/17:0 (TAG). After extraction, the organic phase was transferred to an infusion plate and dried in a speed vacuum concentrator. 1st step dry extract was resuspended in 7.5 mM ammonium acetate in chloroform/methanol/propanol (1:2:4, V:V:V) and 2nd step dry extract in 33% ethanol solution of methylamine in chloroform/methanol (0.003:5:1; V:V:V). All liquid handling steps were performed using Hamilton Robotics STARlet robotic platform with the Anti Droplet Control feature for organic solvents pipetting.

#### MS data acquisition

Samples were analyzed by direct infusion on a QExactive mass spectrometer (Thermo Scientific) equipped with a TriVersa NanoMate ion source (Advion Biosciences). Samples were analyzed in both positive and negative ion modes with a resolution of R_m/z=200_ = 280,000 for MS and R_m/z=200_ = 17,500 for MSMS experiments, in a single acquisition. MSMS was triggered by an inclusion list encompassing corresponding MS mass ranges scanned in 1 Da increments (Surma et al. 2015). Both MS and MSMS data were combined to monitor CE, DAG and TAG ions as ammonium adducts; PC, PC O−, as acetate adducts; and CL, PA, PE, PE O−, PG, PI and PS as deprotonated anions. MS only was used to monitor LPA, LPE, LPE O−, LPI and LPS as deprotonated anions; Cer, HexCer, SM, LPC and LPC O− as acetate adducts.

#### Data analysis and post-processing

Data were analyzed with in-house developed lipid identification software based on LipidXplorer (Herzog et al. 2011, 2012). Data post-processing and normalization were performed using an in-house developed data management system. Only lipid identifications with a signal-to-noise ratio > 5, and a signal intensity fivefold higher than in corresponding blank samples were considered for further data analysis.

The heatmap-plot was generated from z-scores of pmol values of all lipid species by using the Origin software (OriginLab, Northhampton, MA, USA). Principal component analysis identified one of five individual experiments as outliner (Additional file 3: Fig. S2). Thus, four instead of five cohorts were used for further evaluation. An additional file with mol%-values of all lipid species is provided (see Additional file 1; Tab. S1).

#### Calculation of average number of double bonds and chain length

The following equations were used for calculations of double bonds (2) and chain length (3). Average values calculated using these equations refer to the total lipids containing two fatty acids.

The average number of double bonds within a lipid class *DB*_*av*_ was calculated by the following equation

2 $${DB}_{av} = \sum \nolimits_{k =1}^{n} \frac{lip_{n}}{class} DB_n $$

where DB is the number of double bonds of an individual lipid species, *lip* is the amount of the respective lipid species in pmol and class is the pmol amount of the respective lipid class.

The average chain length *CL*_*av*_ was calculated by the following equation

3 $${CL}_{av} = \sum \nolimits_{k =1}^{n} \frac{lip_{n}}{class} CL_n $$

where CL is the number of C atoms of an individual lipid species.

### Statistics

Raw data from fluorescence-anisotropy experiments were processed by Excel (Microsoft office 2013, Redmond, Washington, USA). Ten fluorescence anisotropy measurements of one experiment were pooled and the mean value was calculated. Three individual experiments were performed.

For lipidomics results for independent experiments were performed. For statistical analysis one way-ANOVA was performed, followed by Holm-Šídák test using the Prism V.6 software (GraphPad, La Jolla, CA, USA). Results significantly different from their corresponding control groups are marked by *p < 0.05.

## Results

Changes of the plasma membrane fluidity of commercially available PBMC exposed to cortisol and Ze 117 were assessed by fluorescence anisotropy measurements. For this purpose DPH (1,6-diphenyl-1,3,5-hexatriene) was applied as a fluorescent membrane probe. The tumbling rate of DPH is increased by a decreased membrane viscosity (increased membrane fluidity) causing fluorescence anisotropy (Mitchell and Litman 1998).

**Fig. 2:**
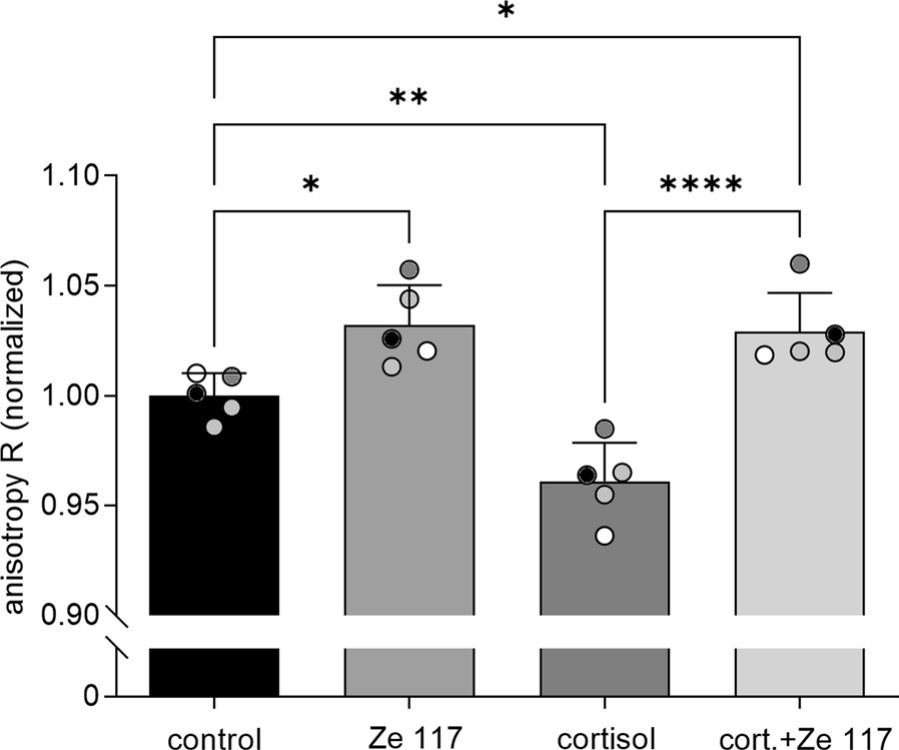
Fluorescence anisotropy of untreated PBMC (control) and after treatment with 1 µM cortisol, 50 µg/ml Ze 117 and combination of 1 µM cortisol with 50 µg/ml Ze 117 using 2.5 µM DPH. Results of five independent experiments are presented as mean ± standard deviation. *Marked values are significantly different from corresponding control with p < 0.05 (**p < 0.01, ***p < 0.001, ****p < 0.0001) determined by one-way ANOVA (n = 5), followed by Holm-Šídák test

PBMC that were pre-treated with 1 µM cortisol for 4 days showed a significant (p = 0.0061) decrease in relative fluorescence anisotropy to 0.96 ± 0.018 compared to the control. An opposite effect was observed after 4 days of incubation with 50 µg/ml Ze 117. Fluorescence anisotropy significantly (p = 0.019) increased to 1.03 ± 0.018 compared to control leading to a significant plasma membrane rigidification (Fig. 2).

The cortisol mediated effect was reversed when PBMC were co-incubated with cortisol and Ze 117.

To further determine what caused membrane fluidity a comprehensive lipidomic analysis of cortisol and cortisol/Ze 117 pre-incubated PBMC. By co-incubation of cortisol with Ze 117 it was investigated whether Ze 117 counteracts a cortisol-induced change in the lipid composition of PBMC.

**Fig. 3:**
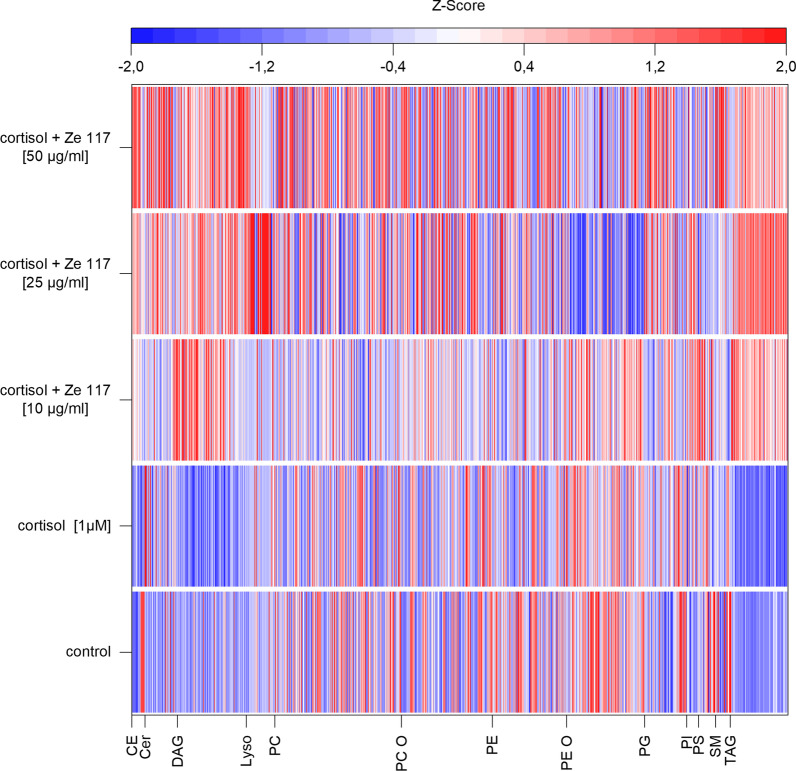
Heatmap of z-scores of all analysed 890 lipid species calculated from average mol%-values generated by lipidomics. Main lipid classes are given on the x-axis (*CE* cholesteryl ester, *Cer* ceramides, *DAG* diacylglycerols, *Lyso* lyso-phospholipids, *PC* phophatidylcholines, *PC O* phosphatidylcholine ethers, *PE* phosphatidylethanolamines, *PE O* phosphatidylethanolamine ethers, *PG* phosphatidylglycerol, *PI* phosphatidylinositol, *PS* phosphatidylserine, *SM* sphingomyelin, *TAG* triacylglycerides)

Lipidome alterations after treatments are visualized in the heatmap plot (Fig. 3). Cortisol treatment downregulated most of the lipids compared to control. Co-incubation with Ze 117 tended to normalize the lipid species to control levels dose-dependently. An exception are storage lipids such as TAG and CE, which are not regulated by cortisol but are considerably increased by co-incubation with Ze 117.

However, this effect was not statistically significant on the single lipid species level apart from a few phospholipid species and mostly storage lipids, such as TAG and CE.

To get a more global impression of the alterations in the lipidome, alterations in the average number of double bonds and the average chain length of the fatty acids within individual lipid classes were looked at instead of the occurrence of individual lipids.

**Fig. 4:**
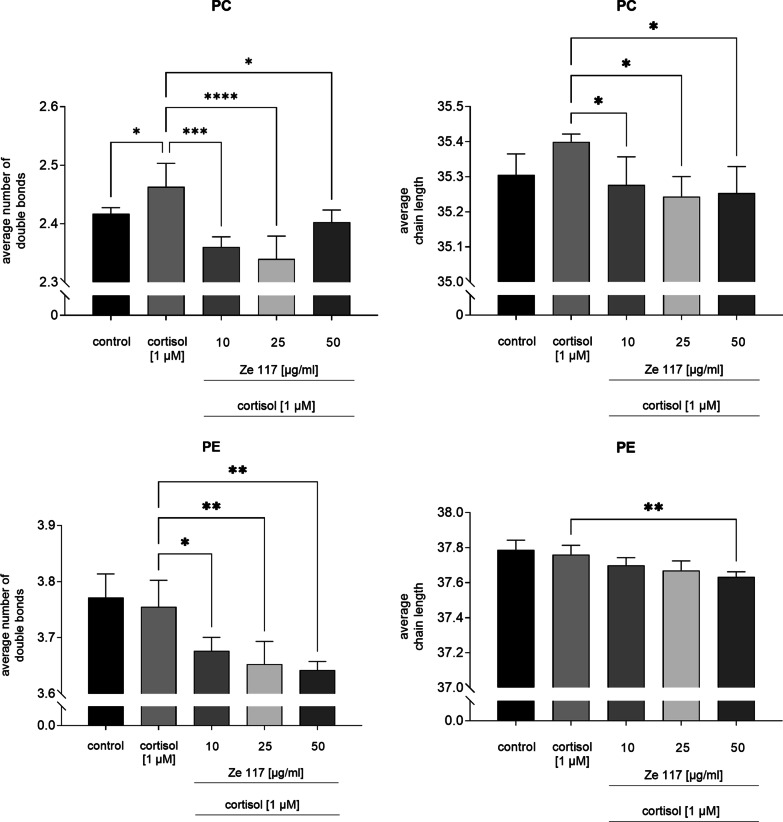
Average chain length and average number of double bonds of phosphatidylcholines (PC) and phosphatidylethanolamines (PE) of PBMC after cortisol and cortisol/Ze 117 preincubation, respectively. For statistical evaluation the cortisol condition was compared to the control. Average chain length and average number of double bonds of cortisol/Ze 117 pre-treated cells were compared to the cortisol condition. *Marked values are significantly different from corresponding control with p < 0.05 (**p < 0.01, ***p < 0.001, ****p < 0.0001) determined by one-way ANOVA (n = 4), followed by Holm-Šídák test

Compared to the control, an increase (p = 0.052) in the average chain length from 35.31 ± 0.06 to 35.40 ± 0.02 and a significant increase (p = 0.0062) in the average number of double bonds from 2.42 ± 0.010 to 2.46 ± 0.04 within phosphatidylcholines (PC) was found for cortisol treated PBMC (Fig. 4). In contrast, the average chain length after co-incubation with 1 µM cortisol and 50 µg/ml Ze 117 was brought to control level, indicating a significant (p = 0.0021) counteracting effect of Ze 117. In lower concentrations up to 10 µg/ml Ze 117 a comparable effect was observed. A clear dose–response relationship is therefore missing. In addition, the average number of double bonds after preincubation with cortisol and 50 µg/ml Ze 117, respectively, was reduced to the control level. Remarkably, with lower Ze 117 concentrations the decrease in the average number of double bonds was more pronounced.

In cortisol pre-treated PBMC both the average chain length and the average number of double bonds of phosphatidylethanolamines (PE) were not affected, compared to control. In contrast, after cortisol/Ze 117 pretreatment the average chain length was reduced dose-dependently and significantly (p = 0.0081) from 37.76 ± 0.05 to 37.63 ± 0.03 compared to cortisol pre-treated cells. Similarly, the average number of double bonds decreased dose-dependently and significantly (p = 0.0019) from 3.76 ± 0.05 to 3.64 ± 0.02.

**Fig. 5:**
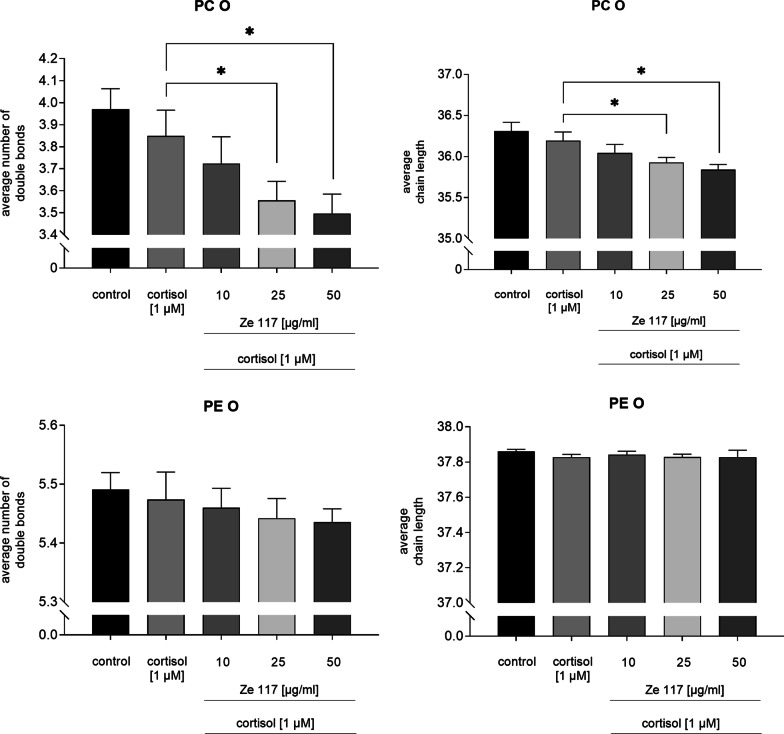
Average chain length and average number of double bonds of phosphatidylcholine ethers (PC O) and the phosphatidylethanolamine ethers (PE O) species after cortisol and cortisol/Ze 117 preincubation, respectively. For statistical evaluation the cortisol condition was compared to the control. Average chain length and average number of double bonds of cortisol/Ze 117 pre-treated cells was compared against the cortisol condition. *Marked values are significantly different from corresponding control with p < 0.05 (**p < 0.01, ***p < 0.001, ****p < 0.0001) determined by one-way ANOVA (n = 4), followed by Holm-Šídák test

Neither cortisol alone, nor co-incubation with Ze 117 had an impact on the average number of double bonds and the average chain length of fatty acids in the phosphatidylethanolamine ethers class (PE O) (Fig. 5).

Cortisol alone did not alter the fatty acid composition in the phosphatidylcholine ethers class (PC O). After co-incubation with Ze 117 a significant (p = 0.0003) and dose-dependent decrease in the average chain length of fatty acids within the PC O class was found. The average chain length decreased significantly (p = 0.0003) from 36.19 ± 0.11 to 35.84 ± 0.07 after co-incubation with 1 µM cortisol and 50 µg/ml Ze 117, compared to cortisol pre-treated cells. The same effect was observed for the average number of double bonds which dose-dependently and significantly (p = 0.0011) decreased from 3.85 ± 0.12 to 3.50 ± 0.09.

**Fig. 6:**
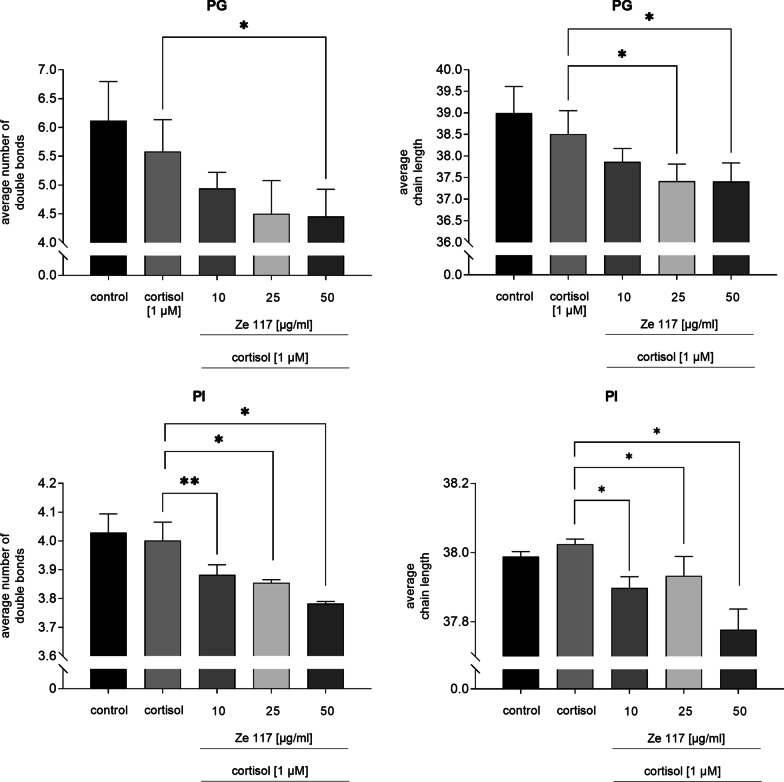
Average chain length and average number of double bonds of the lipid classes of phosphatidylglycerols (PG) and phoshpatidylinositols (PI) after cortisol and cortisol/Ze 117 preincubation, respectively. For statistical evaluation the cortisol condition was compared to the control. Average chain length and average number of double bonds of cortisol/Ze 117 pre-treated cells were compared with the cortisol condition. *Marked values are significantly different from corresponding control with p < 0.05 (**p < 0.01, ***p < 0.001, ****p < 0.0001) determined by one-way ANOVA (n = 4), followed by Holm-Šídák test

As shown in Fig. 6, cortisol alone did not alter the fatty acid composition in the phosphatidylglycerol (PG) class. After co-incubation with Ze 117 a significant and dose-dependent decrease in the average chain length of fatty acids within the PG class was found. The average chain length decreased significantly (p = 0.020) from 38.51 ± 0.55 to 37.41 ± 0.44 after co-incubation with 1 µM cortisol and 50 µg/ml Ze 117, compared to cortisol pre-treated cells. The same effect was observed for the average number of double bonds which dose-dependently and significantly (p = 0.036) decreased from 5.58 ± 0.55 to 4.45 ± 0.47.

The average chain length of fatty acids within the phosphatidylinositol (PI) class did not change after cortisol treatment compared to control cells. Co-incubation with 50 µg/ml Ze 117 induced a significant (p < 0.0001) decrease of the average chain length from 38.02 ± 0.02 to 37.78 ± 0.06, which remarkably was below control level. This was also observed for co-incubation with cortisol and 10 µg/ml Ze 117 and 25 µg/ml Ze 117, respectively, but to a lesser extent. No effect was seen for the average number of double bounds for cortisol treated cells. Co-incubation with Ze 117, however, reduced the average number of double bounds within the PI class significantly (p = 0.0002) and dose-dependently from 4.00 ± 0.06 in cortisol pre-incubated cells to 3.78 ± 0.01 in PBMCs.

**Fig. 7:**
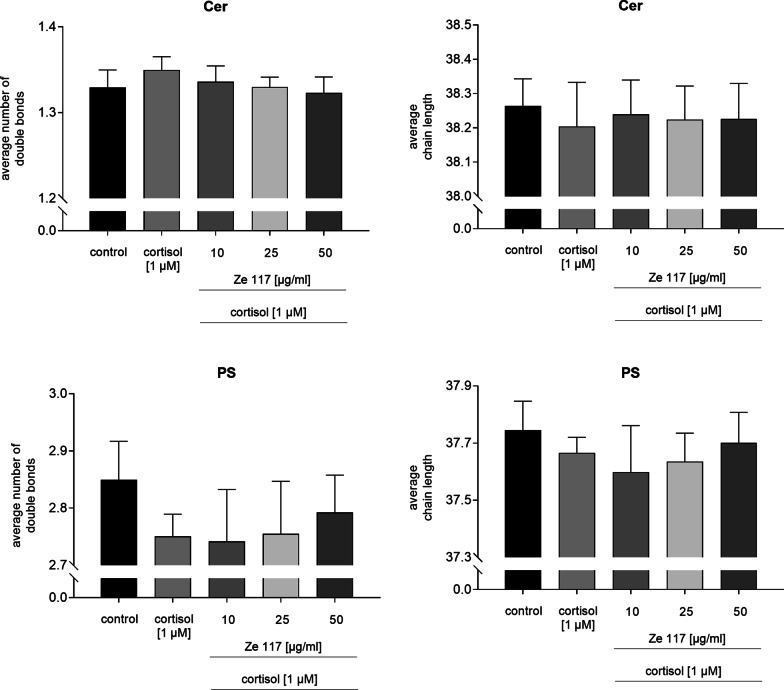
Average chain length and average number of double bonds of the lipid classes of ceramides (Cer) and phoshpatidylserines (PS) after cortisol and cortisol/Ze 117 preincubation, respectively. For statistical evaluation the cortisol condition was compared to the control. Average chain length and average number of double bonds of cortisol/Ze 117 pre-treated cells were compared with the cortisol condition. *Marked values are significantly different from corresponding control with p < 0.05 (**p < 0.01, ***p < 0.001, ****p < 0.0001) determined by one-way ANOVA (n = 4), followed by Holm-Šídák test

Neither cortisol alone nor co-incubation with Ze 117 had an impact on the average number of double bonds and the average chain length of fatty acids of ceramides (Cer) and phosphatidylserines (PS) (Fig. 7).

## Discussion

St. John’s wort extracts have been intensively investigated in several in vitro and in vivo studies for their mechanism of antidepressant action (for review see Schmidt and Butterweck 2015). A variety of potential mechanisms have been discussed to be responsible for the antidepressant activity of SJW. Among these are the effects on 5-HT and β-adrenergic receptors, inhibition of inflammatory cytokine levels or a decrease in brain-derived neurotrophic factors in the rat hippocampus (Schmidt and Butterweck 2015; Valvassori et al. 2018).

Since the stress responsive HPA axis has been implicated in the pathophysiology of depression, several studies have shown modulating effects of SJW extracts on HPA axis function in rats such as a reduced gene expression of genes that control HPA axis function (Butterweck et al. 2001a; b) as well as decreased plasma adrenocorticotropic hormone (ACTH) and corticosterone levels in stressed rats (Butterweck et al. 2001a; b). However, none of these targets mentioned above can fully explain the exact mechanism of antidepressant action of SJW extract.

Increasing evidence suggests that stressors such as cortisol affect the biophysical properties of the plasma membrane by causing changes in membrane lipid composition (Tracey et al. 2018). Even small changes in lipid composition of plasma membranes have significant consequences on the modulation of intracellular signaling pathways (Rhode et al. 2009; Vigh et al. 2007).

It has been shown in rainbow trout liver that plasma membrane fluidization caused by cortisol stress involves changes in the lipid topography of the plasma membrane (Dindia et al. 2013). As hypothesized by Vigh et al. (2007) membrane lipids could not only be ‘targets of stress’ but also ‘sensors’ which enable stress responses through a general effect on membrane fluidity.

Since membrane lipids seem to be involved in the pathogenesis of depression (for review see Müller et al. 2015) they also could present an important target in the antidepressant action of SJW, thus providing a further link to the so far only incompletely explained mechanism of action. To further study the involvement of membrane lipids in the mechanism of action of SJW, fluorescence anisotropy measurements as well as lipidomics were performed in the present study.

The plasma membrane consists mainly of different phospholipids, which are arranged in a bilayer (van Meer et al. 2008). The polar, hydrophilic head groups are on the outside and the fatty acid residues are on the inside of the bilayer. In fact, plasma membrane fluidity strongly depends on the chain length and the number of double bonds of the fatty acid moieties of phospholipids. Longer chain lengths result in stronger van der Waal’s interactions and therefore to an increased rigidity of the plasma membrane. Cis-configured double bonds are responsible for curvatures in unsaturated fatty acids. If phospholipids contain fatty acids with more cis-double bonds, the resulting curvatures increase and van der Waal’s interactions between neighbouring acyl chains decrease, leading to a more fluid membrane. In contrast, pure saturated fatty acids can be packed more tightly leading to a more rigid plasma membrane (Mocking et al. 2018).

Recently, we investigated (Keksel et al. 2019) the effect of cortisol on plasma membrane fluidity of C6 glioblastoma cells by fluorescence anisotropy measurements using also DPH (1,6-diphenyl-1,3,5-hexatriene) as a probe. Membrane fluidity refers to the motion of the membrane bilayer and is mainly dependent on cholesterol content, the phospholipid composition and the saturation and chain length of the fatty acid moieties (Ballweg and Ernst 2017; Zhang et al. 2020). Alterations in membrane fluidity are therefore a strong indicator of changes in the lipid composition of the plasma membrane. Our working group (Keksel et al. 2019) showed that plasma membrane fluidity in C6 glioblastoma cells increased when exposed to the stress hormone cortisol. An opposite effect was seen for C6 glioblastoma cells incubated with the St. John’s wort extract Ze 117. The cortisol mediated effect was reversed when C6 glioblastoma cells were co-incubated with cortisol and Ze 117 (Keksel et al. 2019).

Although the C6 rat glioma cell line is widely used in neuroscience, it was of interest for this study to use a cell line which is better suited to investigate changes in cellular lipids. Several in vitro studies have previously demonstrated a changed membrane fluidity after chronic preincubation with glucocorticoids using different cell types, e.g. lymphocytes (Tolentino et al. 1991), HeLa cells (Johnston and Melnykovych 1980), and intestinal membranes (Brasitus et al. 1987).

Peripheral blood mononuclear cells (PBMC) play critical roles in immune response and metabolism. It has been shown that several parallel responses in the brain and PBMC exist such as alterations of metabolism and disturbances in the main neurotransmitter and hormonal systems (Costi et al. 2021; Li et al. 2014). Since the use of brain biopsy samples of patients is unfeasible for studying lipid alterations, PBMC might be a practical alternative cellular system to investigate changes in lipid composition and metabolism. Interestingly, Tolentino et al. (1991) demonstrated in a previous study that cortisol directly increased lymphocyte membrane fluidity as measured by the polarization of fluorescence from the membrane-associated probe DPH in peripheral blood lymphocytes. Membrane fluidity increased in vitro after short- or long-term cortisol exposure (Tolentino et al. 1991). This is in so far of importance for the present study since the majority of the PBMC population mainly consists of lymphocytes.

Evidence for alterations in the lipid composition of brain tissue after antidepressant therapy has already been shown in several in vivo studies (Pinto et al. 2022). Plasma (Pinto et al. 2022) or blood cells such es erythrocytes (Edwards et al. 1998) have therefore been used in clinical trials to monitor progression of major depression or antidepressant treatment effect. In the present experiments Ze 117 also showed a membrane rigidity-increasing effect in PBMC, counteracting a higher membrane fluidity after cortisol treatment. Since DPH integrates in the deep hydrophobic core of the acyl chain regions of the phospholipid bilayer, it marks changes in the fatty acid moieties, compared to probes such as laurdan, which integrates into the glycerol backbone of phospholipids.

We also demonstrated in previous experiments that the effect of Ze 117 on the membrane fluidity of C6 glioblastoma cells correlated with a changed molecular structure of fatty acid moieties of membranous phospholipids (Keksel et al. 2019). A plasma membrane rigidification in murine microglia cells was also described by Kraus et al. (2007) after a 24 h incubation with a non-commercial SJW extract.

Numerous other studies have highlighted a relationship between major depressive disorders (MDD) and the lipidome (Kim et al. 2018; Lin et al. 2010; Liu et al. 2016; Mocking et al. 2018).

Mocking et al. (2018) concluded in a review regarding fatty acids in psychiatric disorders, that fatty acid metabolism forms a complex neurometabolic network that seems to alter the vulnerability of psychiatric diseases. Knowles et al. (2017) found that alterations in the lipidome are not necessarily secondary to the manifestation of MDD, but rather share etiology with the illness. The authors concluded that lipids, their fatty acid moieties and their molecular pathways are promising candidates when attempting to improve diagnostics and treatment efforts in MDD. Based on these findings mentioned above, the fatty acid composition of phospholipids was further investigated in the present study by a comprehensive lipidomic analysis of PBMC pre-incubated with cortisol and Ze 117. The heatmap data clearly show that cortisol as well as co-incubation of cortisol with Ze 117 caused qualitative and dose dependent changes in the lipidome. Significant differences at the level of individual phospholipids or cholesterol esters, which could explain the changes in membrane fluidity, were not detected. However, it has to be noted that statistical methods how to handle the complexity of lipid data are discordant in the literature (Mocking et al. 2012) and it is important to take into account that lipid species in membranes act not as single molecules but as a collective (Liebisch et al. 2019). It therefore seems more appropriate to look at more global changes in the lipidome rather than at the level of individual lipids. In the current study we therefore focused on specific characteristics such as lipid unsaturation (double bonds) and chain lengths analysis of fatty acid residues to characterize the cellular phenotype, a method, which is frequently used in lipidomic data analysis (Lin et al. 2021). The effects of cortisol and the cortisol/Ze 117 combination, respectively, on the average chain length and the average number of double bonds of fatty acid residues were therefore analyzed for several lipid classes. The evaluation was based on quantitative values of each lipid species relative to total amounts of the respective lipid classes assessed by mass spectrometry analysis. In lipidomic studies both chain lengths and double bonds of fatty acids are described as robust and resilient parameters (Chitraju et al. 2013; Thiele et al. 2019). Thus, small differences indicate changes in the lipid metabolism of the cells. Compared to the control, an increase in both the average chain length and the average number of double bonds within phosphatidylcholines (PC) was found for cortisol treated PBMC. In contrast, the average chain length after co-incubation with 1 µM cortisol and 50 µg/ml Ze 117 was brought to control level, indicating a significant counteracting effect of Ze 117. A similar counteracting effect of Ze 117 compared to cortisol-treated cells was observed for the average chain length of phosphatidylinositols (PI). The decreased chain length after Ze 117 preincubation would suggest an increased membrane fluidity. However, in this work the membrane fluidity decreased after Ze 117 preincubation. This suggests that the reduced number of double bonds by Ze 117 dominates the effect on membrane fluidity.

In a study in rats by Oliveira et al. (2016), chronic unpredictable stress led to a significant decrease in monounsaturated fatty acids in different brain regions. At the same time, chronic unpredictable stress led to an increase in fourfold unsaturated fatty acids. This is consistent with the results of the current work, in which cortisol-induced stress in PBMC increased the average number of double bonds and at the same time the antidepressant Ze 117 had an opposite effect. A similar effect was observed in a clinical study by Otoki et al. (2017) who investigated seasonal changes in the occurrence of fatty acids bound to phospholipids, triacylglycerols and cholesterol esters in the plasma of patients with winter depression. The authors observed a non-significant but continuous increase in phospholipids of all long chain poly unsaturated acids (LC-PUFA) during winter season.

While alterations in membrane lipid composition have been shown in several animal models of depression or in patients with MDD, only few studies exist that investigated the effect of antidepressant treatment on lipid profiles. Most of these studies were performed in rodents or primates treated with different classes of antidepressants (for review see Pinto et al. 2022) and only very few data are available in humans. In a study on juvenile macaques long-term treatment with the antidepressant fluoxetine caused substantial lipidome alterations in the brain (Tkachev et al. 2021). Interestingly, a decrease in polyunsaturated fatty acids (PUFAs) was observed in almost all lipid classes in the brain following fluoxetine administration (Tkachev et al. 2021). A similar result has recently been found for human depressive patients who were treated with the selective serotonin reuptake inhibitors (SSRI) citalopram/escitalopram (MahmoudianDehkordi et al. 2021). After treatment the plasma samples of remitted patients showed significant changes in the lipidome compared to patients with treatment failure: e.g., phosphatidyl choline (PC) species containing saturated or monounsaturated fatty acyl chains tended to increase (PC species containing ≤ 3 total double bonds), whereas PC species containing PUFAs (species containing ≥ 4 total double bonds) were reduced (Edwards et al. 1998). Such effects did not occur in the treatment failure group. The authors concluded that among others remodeling of fatty acid chains may play a role in SSRI treatment response (Edwards et al. 1998). While all these data are promising, further studies in humans are necessary in which the plasma lipid profile of placebo treated depressed patients should be compared with the lipid profile of patients treated with antidepressants to allow a better assessment of particular lipid biomarkers.

## Conclusions

Membrane lipid alterations seem to play an important role in the development of depressive disorders. The data generated in the present study by fluorescence anisotropy measurements demonstrate that cortisol and Ze 117 show opposite effects on plasma membrane fluidity. Considering the results of the lipidomic analysis, altered plasma membrane fluidity might be caused by changes in the fatty acid composition of the lipidome. The lipidome might therefore represent a novel target for the antidepressant action of SJW extracts.

To our knowledge, our current study is the first investigating the effects of a SJW extract on the cellular lipid profile using PBMC. Considering data from the literature discussed above it seems that the SJW extract Ze 117 exerts effects on the cellular lipid profile comparable to antidepressants such as fluoxetine or citalopram. To support this hypothesis, further studies comparing the effects of Ze 117 and standard antidepressants on changes in the lipid profile in rodent brain and plasma samples are currently under investigation.

## Supplementary Information

**Additional file 1: Table S1.** Excel spreadsheet with raw lipidomics data.

**Additional file 2: Figure S1.** Representative HPLC chromatogram of the St. John’s wort extract Ze 117.

**Additional file 3: Figure S2.** Principal component analysis: 2D Scatter plot with the principal components 1 and 2 of all 25 samples. The following five conditions were included in each experimental cohort: Control (o), cortisol (+) and cortisol with 10 µg/ml Ze117 (*), 25 µg/ml Ze117 (*) and 50 µg/ml Ze117 (*), respectively. A clustering of the samples preincubated with Ze117 was observed, which is marked with a red circle. The samples of the control and cortisol condition also form a cluster, which is circled in pink. All samples of cohort 2 form an own cluster (blue circle). Thus, cohort 2 was identified as outliner and removed from further data evaluation.

## Data Availability

The datasets used and/or analysed during the current study are available from the corresponding author on reasonable request.

## Abbreviations

Cer: Ceramides
FA: Fatty acids
PBMC: Peripheral blood mononuclear cells
PC: Phosphatidylcholines
PC O: Phosphatidylcholine ethers
PE: Phosphatidylethanolamines
PE O: Phosphatidylethanolamine ethers
PG: Phosphatidylglycerols
PI: Phosphatidylinositols
PUFA: Polyunsaturated fatty acids
SJW: St. John’s wort
