# Supplementary figures and images for: Impact of St. John’s wort extract Ze 117 on stress induced changes in the lipidome of PBMC
Source: Mol Med. 2023 Apr 7;29:50. doi: 10.1186/s10020-023-00644-3 (PMC10082490; doi:10.1186/s10020-023-00644-3)

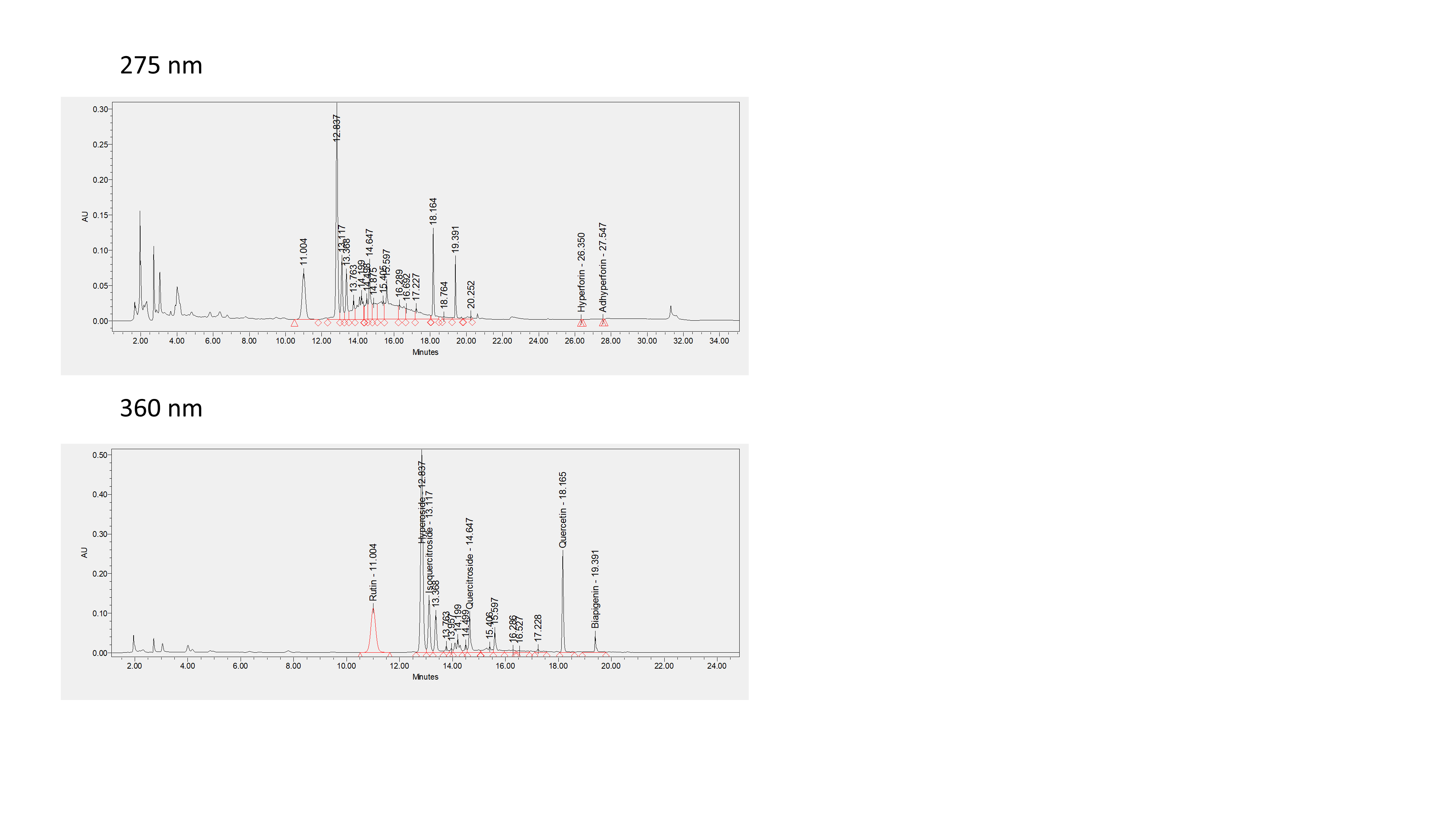

Supplement: Supplementary file 2 — Additional file 2: Figure S1. Representative HPLC chromatogram of the St. John’s wort extract Ze 117. [file 10020_2023_644_MOESM2_ESM.tif]

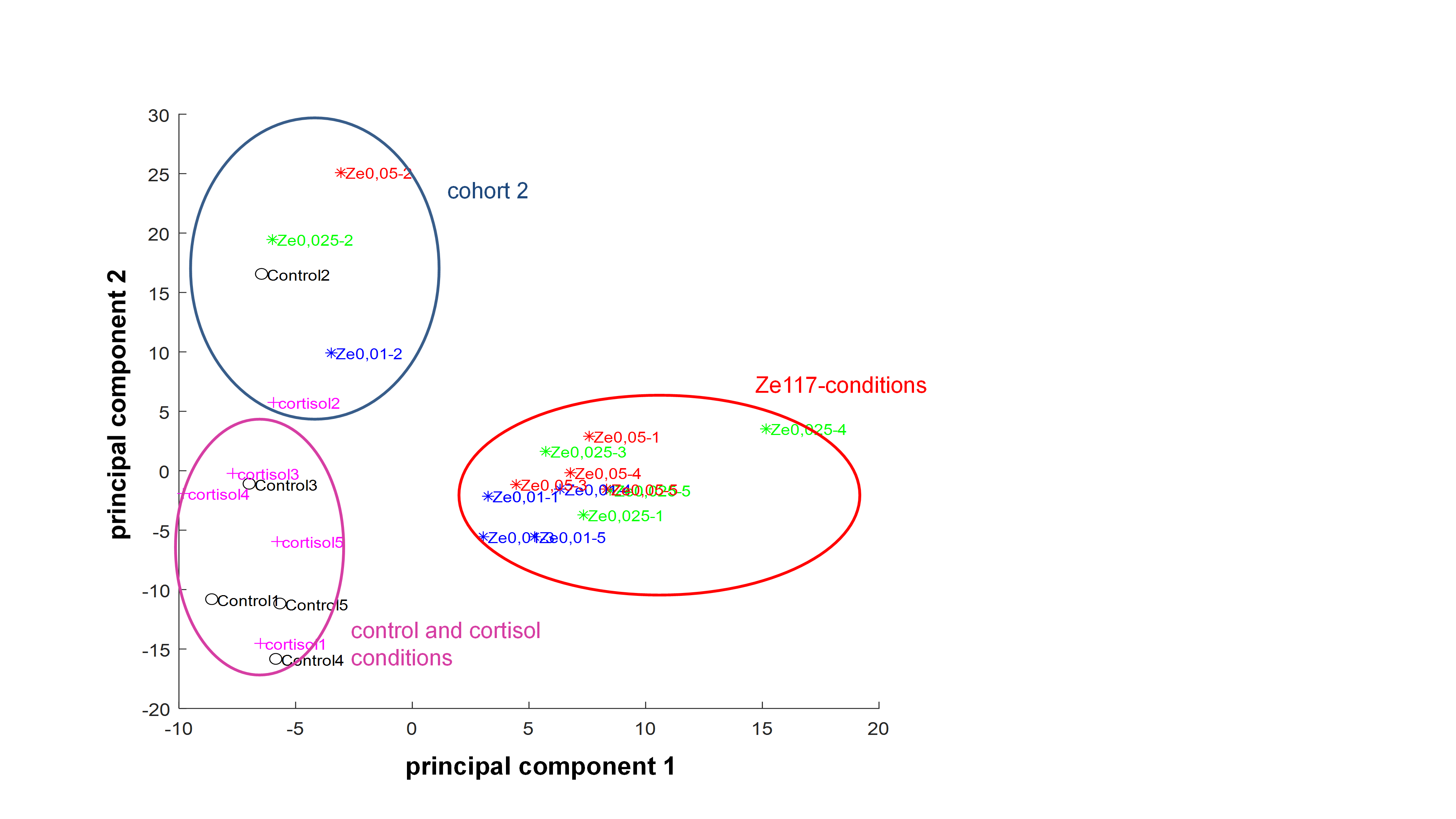

Supplement: Supplementary file 3 — Additional file 3: Figure S2. Principal component analysis: 2D Scatter plot with the principal components 1 and 2 of all 25 samples. The following five conditions were included in each experimental cohort: Control (o), cortisol (+) and cortisol with 10 µg/ml Ze117 (*), 25 µg/ml Ze117 (*) and 50 µg/ml Ze117 (*), respectively. A clustering of the samples preincubated with Ze117 was observed, which is marked with a red circle. The samples of the control and cortisol condition also form a cluster, which is circled in pink. All samples of cohort 2 form an own cluster (blue circle). Thus, cohort 2 was identified as outliner and removed from further data evaluation. [file 10020_2023_644_MOESM3_ESM.tif]
